# Supplementary material for: Comparative iTRAQ proteomic profiling of sweet orange fruit on sensitive and tolerant rootstocks infected by ‘Candidatus Liberibacter asiaticus’
Source: PLoS One. 2020 Feb 14;15(2):e0228876. doi: 10.1371/journal.pone.0228876 (PMC7021301; doi:10.1371/journal.pone.0228876)
Supplement: Attachment — Submitted filename: Response to Reviewers.pdf [file pone.0228876.s005.pdf]

UF UNIVERSITY of FLORIDA

Institute of Food and Agricultural Sciences
Citrus Research and Education Center

700 Experiment Station Road
Lake Alfred, FL 33850-2299
863-956-1151
863-956-4631 Fax
Website: www.crec.ifas.ufl.edu

17 January 2020

Dear Dr. Huang,

Thank you and the reviewers very much for your review and good suggestions. We revised the manuscript according to the concerns and suggestions. Please see our detailed responses point by point, below.

For Reviewer #1

Suggestion Line 24 ‘incurable’ is not accurate. In some studies and reports HLB affected trees could be cured by applying antibiotics or high temperature treatment. The main challenge of HLB is that there has not been any robust controlling method that could solve the problem once for all.

Reply: We replaced “incurable’ with ‘destructive’ in line 23 of “Manuscript_PONE-D-19-33708”.

Suggestion Line 36 and 39 should be ‘jasmonate signaling’; Line 47 delete ‘economically’; Line 49 ‘non-culturable’ is controversial and should be deleted, some study has reported the culture of the bacteria though at very low titer.

Reply: Have done in lines 35, 37, and 44 of “Manuscript_PONE-D-19-33708”; ‘non-culturable’ was replaced with more commonly recognized terminology ‘fastidious’ in line 46.

Suggestion Line 54 What does ‘non-specific’ mean? Do other disease have similar symptoms? If yes why it was still ‘readily distinguishable’? Should be explained or modified.

Reply: We deleted the “non-specific” sentence, for the following sentences described the characteristics of symptomatic fruit.

Suggestion Line 55-56 Do symptomatic fruits always abscise prematurely? If not, 'often' should be added in front of abscise; Line 62-63 'fruit contains lower percentage of soluble solid content and has lower soluble solid content to titratable acidity ratio'.

Reply: Have done.

Suggestion Line 63 'amount of' should not followed by non-countable noun

Reply: Change 'amount of' to 'concentration of'.

Suggestion Line 64, lack an 'and'; Line 71 'most of them'; Line 78 delete 'phenomena' and 'even', and use past form 'showed'; Line 82 "yet they have not yet been"; Line 85 delete 'even', should denote whether the HLB-sensitive scion/rootstock is CaLas-infected; Line 89 'tolerant and sensitive citrus rootstocks'; Line 91 Better modified to be 'are unevenly distributed' and 'could induce similar or different'

Reply: Have done.

Suggestion Line 93 'Due to spreading quickly and the difficulties to control HLB' should be rewritten; Line 95-97, 'Because' and 'therefore' used together. There seemed to be no strong causal relationship here, and sentence had better be re-structured

Reply: We rewrote it.

Suggestion Line 163 'characteristics'

Reply: Have done in line 156 of "Manuscript_PONE-D-19-33708".

Suggestion Line 176 Has statistical analysis been done to test the size and weight different? Are there any DE proteins related with fruit quality alteration? There is no discussion on this area at all, then why choosing fruit rather than leaves or other tissues as experimental material.

Reply: We did perform statistical analyses to test the size and weight differences; the results are mentioned in lines 165-170 of "Manuscript_PONE-D-19-33708". Just like the leaves and roots, the development and maturity of fruit is under the HLB stress. Many studies have shown the differentially expressed genes or proteins in leaves and roots. However, we did not know if there

are *Ca*Las-sensitive or –tolerant pathways involved in fruits. This is the reason for choosing fruit as the experimental material. We added this sentence to the introduction: "There is very little known about the proteomic differences in fruit pulp tissues as a consequence of HLB, and nothing is known on the impact of using more tolerant rootstocks on the fruit proteome".

Suggestion Line 178 'proteomic profile'
Reply: Have done in line 171 of "Manuscript_PONE-D-19-33708".

Suggestion Line 182-183 Different numbers of DE proteins from that described in Abstract
Reply: We checked the data carefully and corrected the numbers of DE proteins. For example, we said 573 DE proteins in the symptomatic fruit on 'Swingle' in Abstract part, which equaled with 495 DE fruit proteins found only in the symptomatic fruit on 'Swingle' and 78 DE proteins shared in fruit on both rootstocks in Results part.

Suggestion Line 263 'serious destructive' has grammar error. 'uncured disease' not appropriate here
Reply: We rewrote it.

Suggestion Fig 1. The go terms had better be put in the graph instead of the number IDs which would make the graph easier to read.
Reply: Have done.

Suggestion There are still a few other grammar mistakes should be revised.
Reply: We did our best to correct the other grammar mistakes.

For Reviewer #2
Suggestion As shown, the current tables in the manuscript only presented a small part of the differentially expressed proteins, and all other data were enclosed in the supplementary, that restrict the readers to get a full view of the result. A table is fine to present a small number of

proteins, but quite limited for hundreds of proteins. Since abundant of differentially expressed proteins were obtained from the comparative proteomic analysis, a more convenient and intuitive interface is quite necessary to present your data, such as pathways combined with hot-map.
Reply: We used MapMan to analyze the differentially expressed proteins in biotic stress. The new figure (Figure 1A) provides a more comprehensive view of the results.

Suggestion It is better to use only the name of rootstock instead of “HLB tolerant” or “HLB sensitive” throughout the manuscript after the first introduction of material background.
Reply: Have done in most places, though kept this designation in the Conclusion section for clarity.

Suggestion Line 28-31: Confused long sentence. Short sentence can be more understandable, e.g. "same materials without CaLas infection were used as controls”.
Reply: Have done.

Thanks again

Sincerely yours,

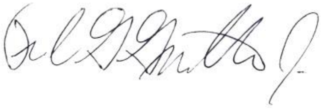

Fred G. Gmitter Jr.
UF Research Foundation Professor
Professor of Citrus Breeding and Genetics
Email: fgmitter@ufl.edu
